# Predicting Tumor Sensitivity to Chemotherapeutic Drugs in Oral Squamous Cell Carcinoma Patients

**DOI:** 10.1038/s41598-018-33998-4

**Published:** 2018-10-19

**Authors:** Beaulah Mary Robert, Muralidharan Dakshinamoorthy, Brindha Ganapathyagraharam Ramamoorthy, Muthu Dhandapani, Radhiga Thangaiyan, Ganesan Muthusamy, R. Madhavan Nirmal, Nagarajan Rajendra Prasad

**Affiliations:** 1 Department of Biochemistry and Biotechnology, Annamalai University, Annamalainagar, 608 002 Tamilnadu India; 2 School of Computing, SASTRA Deemed University, Tirumalaisamudram, Thanjavur, 613401 Tamilnadu India; 3 AVVM Sri Pushpam College, Thanjavur, 613503 Tamilnadu India; 4 Department of Oral and Maxillofacial Pathology, Annamalai University, Annamalainagar, 608 002 Tamilnadu India

## Abstract

Oral Squamous Cell Carcinoma (OSCC) patients respond poorly to chemotherapy. We analyzed the expression of 11 drug response-related genes in 31 OSCC biopsies, collected prior to any treatment, using custom-designed PCR array. Further, we investigated the drug response pattern of selected anticancer drugs by BH3 (Bcl2 Homology-3) profiling in the primary cells isolated from OSCC tissues. Then, we correlated the results of drug-response gene expression pattern with apoptotic priming to predict tumor response to chemotherapy. The best performing drug (BPD) and response differences (RD) between the drugs were identified using statistical methods to select the best choice of drug in a personalized manner. Based on the correlation, we classified OSCC tumors as sensitive (13 tumors), moderately responsive (16 tumors) or resistant (2 tumors) to chemotherapy. We found that up-regulation of genes linked with drug resistance facilitates survival of tumor samples, which was revealed by the percentage of apoptotic priming. Moreover, we found that paclitaxel-induced 40–45% apoptotic priming compared to other drugs. Average response difference (RD) analysis showed that 80% of tumors responded well to paclitaxel as compared to other drugs studied. Therefore, gene expression analysis with BH3 profiling reveals drug sensitivity that could be translated for drug selection before treatment.

## Introduction

Oral cancer is a major public health issue, which is responsible for 3–10% of cancer mortality worldwide. The Indian subcontinent accounts for one-third of the world oral cancer burden and the oral cancer ranks among the top three types of cancer in India^1,2^. Annually 130,000 people succumb to oral cancer in this country which translates into approximately 14 deaths per hour^3^. Oral cancers include malignant neoplasms found on the lip, floor of the mouth, cheek lining, gingiva, palate or tongue. Oral Squamous Cell Carcinoma (OSCC) account for more than 90% of all oral malignant lesions^4,5^. It has been demonstrated that a large number of OSCC patients show poor response to chemotherapeutic drugs^6–8^. The clinical drug response of a cancer sub-type seems to be mediated by several mechanisms. These drug response mechanisms include overexpression of membrane transporters effluxing anticancer drugs from the cells, activation of DNA repair enzymes, defects in proteins involved in cell cycle analysis and apoptosis and activation of cytosolic drug detoxification^9–12^.

Ethnic, genetic and epigenetic dissimilarities within clonal populations can determine whether a specific drug combination will benefit a patient or instigate resistance^13^. A significant challenge in various fields of medicine is to assign a drug that will be advantageous^14^. In oncology, this decision has generally been driven by the anatomic location and histology of the tumor. For some time, therapeutic decision making was assisted by cytogenetics, immunohistochemical and flow cytometric analysis of cell surface antigens^14^. However, the forecasters, such as histological grade and lymph node status, would often fail to classify precisely their clinical behavior^15^. Recently, the availability of techniques of gene expression profiling has allowed establishing the relationship between gene expression and drug sensitivity in tumor cells^16^. In the clinical setting, an array of genes has been identified as potential predictive markers of drug activity and their use could be progressively implemented for drug selection in patients receiving chemotherapy, thus allowing more rational and individualized treatments^17^. This type of approach has been initiated with *in vitro* systems by the National Cancer Institute (NCI) in Bethesda, Maryland (USA) and is pursued by a growing number of public and private laboratories around the world^17^. Although extensive research has been performed under *in vitro* conditions concerning drug response and many of the mechanisms have been characterized, translating this to the clinic still represents a major conceptual and technical challenge. Hence, an additional approach that identifies drug sensitivity could significantly advance the clinical management of tumors such as OSCC. Analyzing the expression patterns of genes related to drug response in tumor samples along with evaluating drug response at the cellular level using cell-based assays will assist in drug selection in a personalized manner in order to manage OSCC.

The BCL-2 family proteins are known to control the apoptosis and classified into pro-apoptotic and anti-apoptotic members^18^. Recently, a new functional assay, BCL2-homology domain 3 (BH3) profiling, was reported, and this profiling could predict drug sensitivity in primary tumor samples^19^. The BH3 profiling is a potentially powerful technique to measure early changes in net pro-apoptotic signaling in mitochondria (“apoptotic priming”) induced by chemotherapeutic agents^14^. BH3 profiling interrogates the BCL-2 family of proteins that regulates commitment to the mitochondrial pathway of apoptosis in response to most chemotherapeutic agents^20^. BH3 peptides are convenient, titratable components that can be exploited to systematically study mitochondrial readiness to undergo apoptosis^21^. Thus, BH3 profiling based on the drug’s ability to initiate apoptosis priming can be used to predict the cytotoxic response of cancers to chemotherapeutics before chemotherapy is administered.

There is a significant lack of scientific investigation correlating expression pattern of genes involved in drug response with cell-based experiments to precisely forecast tumor sensitivity towards anticancer drugs. To satisfy this unmet need, we correlated certain drug-response related gene expression patterns with the % apoptotic priming in cancer cells isolated from a cohort of OSCC samples. In this study, we employed a qRT-PCR array to study the expression of certain multidrug resistance (MDR)-linked genes and correlated this with the results of BH3 profiling (percent of apoptotic priming) in order to understand the drug response pattern in a cohort of 31 OSSC tumor specimens.

## Methods

### Tumor samples

Fresh oral tumor biopsies were obtained by routine resections after patients signed an informed consent from Rajah Muthiah Dental College, Annamalainagar. Patients who had already undergone chemotherapy or radiation therapy were excluded from this study. The obtained tumor samples were divided into two parts; (i) for the study of MDR gene expression analysis and (ii) for the analysis of BH3 profiling. For MDR related gene expression studies the tumor biopsies were transferred to sample collection tubes containing RNAlater stabilization reagent. For BH3 profiling the primary tumor cells were isolated and cell viability was assessed by a trypan blue dye exclusion test before actual experiments were performed (Supplementary Fig. 1).

All experiments were performed in accordance with relevant guidelines and regulations. The study was approved by the Institutional Human Ethics Committee of Annamalai University, Annamalainagar (IHEC/0006/2015). The informed consent was obtained from all subjects. Clinical data and tumor samples were collected according to the approved ethical guidelines.

### Treatment

Tissue samples were collected in a sterile tube containing serum-free medium with 1% penicillin-streptomycin-fungizone. The samples were then finely minced by surgical blades after washing with saline. Finely minced tissues were incubated at 37 °C with collagenase for 3–5 h. After incubation, collagenase activity was blocked by the addition of medium with 10% FBS. The solution was then centrifuged at 2000 rpm for 6 min. The cell pellets were resuspended in complete medium and incubated at 37 °C in a 5% CO2 incubator. Equal concentrations of drugs (1 μM) i.e., paclitaxel, doxorubicin, daunorubicin, vincristine and vinblastine were treated in the isolated primary tumor cells (4 × 10^4^ cells/well) for 4 h and the % apoptotic priming was analyzed using BH3 profiling method.

### RNA isolation, quantification and gene expression analysis

The total RNA content of the cells was isolated from tumor tissues using an RNeasy mini kit (Qiagen, India) according to the manufacturer’s instructions and stored at −80 °C until used for gene expression by qRT-PCR arrays. The purity of the freshly isolated total RNA was measured by Nanodrop (Thermo Scientific) and total RNA was employed for the gene expression analysis. The cDNA was reverse transcribed from RNA using a First Strand cDNA Synthesis Kit (Qiagen reverse transcriptase kit). The relative expression pattern of genes related to drug resistance (ABCB1, ABCC1, ABCC2, ABCC3, ABCC5 and ABCG2), apoptosis (TP53), transcription factors (STAT5B and LRP1) and cell cycle regulation (CDKN1A and CDK2) were analyzed by a PCR array using RT^2^ real-time SYBR Green PCR master mix (Qiagen) on an Eppendorf RealPlex instrument. The fold changes of gene expression were analyzed and plotted as clustergram using the SA Biosciences PCR array data analysis online tool (saweb2.sabiosciences.com/pcr/arrayanalysis.php). Relative gene expression (RQ) was calculated using 2^−∆∆Ct^.

### BH3 profiling

The BH3 profiling was performed in tumor tissue samples using BIM peptide (BCL-2-interacting mediator of cell death) to predict drug sensitivity. The BIM peptide was synthesized by the Fmoc solid-phase peptide synthetic strategy and characterized by HPLC and mass spectroscopy (Supplementary Figs 2 and 3). Single suspension of various anticancer drug-treated cells were analyzed for mitochondrial priming using 8.5 μL of digitonin/dye cocktail (4X) composed of 1 µM JC-1, 40 µg/mL oligomycin, 20 mM 2-mercaptoethanol, and 0.01% digitonin (w/v) in TEB (300 mM trehalose, 10 mM HEPES-KOH [pH 7.7], 80 mM KCl, 1 mM EGTA, 1 mM EDTA, 0.1% BSA, 5 mM succinate) for pore formation for the entrance of BH3 peptide into the cell. Then, 15 μl of BIM peptide (1 μM) in TEB was added to each well of a 96-well plate. After 16 h incubation with BIM peptide, the mitochondrial priming was analyzed using a spectrofluorometer (Tecan Infinite Pro, Austria) at an excitation of 545 nm and emission of 590 nm. The percentage loss of ψm for each anticancer agent was calculated by normalization to the solvent only control dimethyl sulfoxide (DMSO) (0% depolarization) and the positive control carbonyl cyanide-p-trifluoromethoxyphenylhydrazone (FCCP) (100% depolarization).

### Statistical analysis

All analyses were carried out using measurements taken in triplicate. Statistical analyses were performed by one-way ANOVA to determine significant differences between groups at P < 0.05. Pearson correlations were tested for significance (by two-tailed unpaired t-test) at the same confidence limit. The Statistical Package, IBM SPSS (Version 21), R programming version 3.3.2 and Microsoft Excel 2007 (Roselle, IL) were used for the statistical and graphical evaluations. To apply linear prediction to the dataset, R programming version 3.3.2 was used. To indicate the response to the drugs, Equations (1 and 2) were applied. The equations to find the Best Priming Drug (BPD) and Response Difference (RD) between BPD and each of the other drugs were analyzed as given below.

1 $${\rm{BPD}}=arma{x}_{i{\in }\{1,\mathrm{2..31}\},j{\epsilon }\{1,\mathrm{2..5}\}}(Drug\_Response[i,\,j])$$

2 $${{\rm{RD}}}_{{{\rm{ij}}}_{{\iota }{\in }}\{1,\mathrm{2..31}\},j{\in }\{1,\mathrm{2..5}\}}=Response(BP{D}_{i})-Drug\_Response[i,\,j]$$

where, *i* is the index of tumors and *j* is the index of drugs (Spplementary Scheme 1).

F-test (variance test) was conducted to know whether the MDR-linked gene expression and % apoptotic priming were influenced by gender difference (Supplementary Table 2). The partition around medoids (PAM) algorithm was used to cluster and classify the tumor samples based on their gene expression pattern and % apoptotic priming. This algorithm minimizes the average dissimilarity of tumor samples compared with the closest selected tumors. The quality of clustering was improved by exchanging the selected tumor with unselected objects.

## Results and Discussion

**Figure 1:**
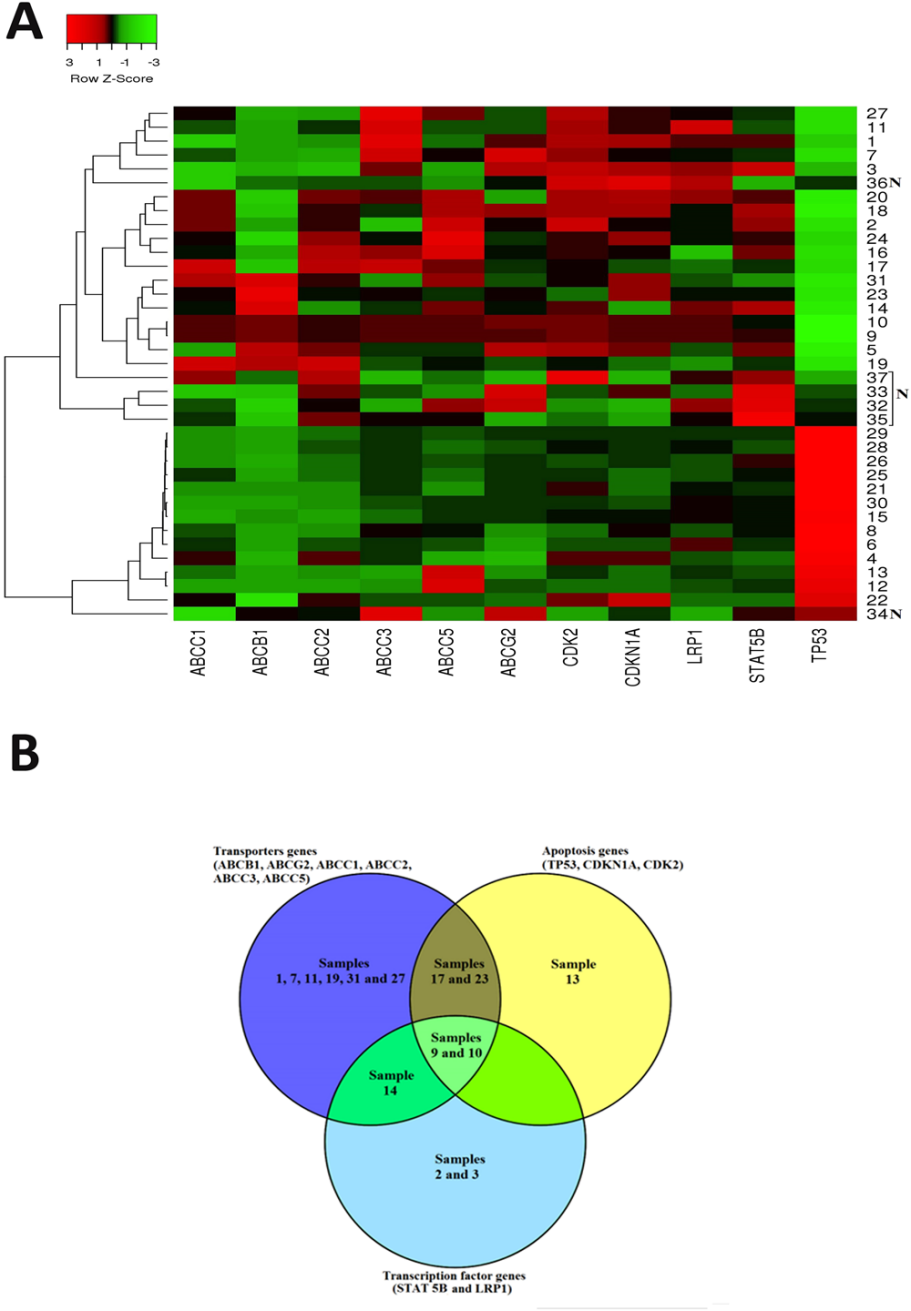
Expression levels of drug response genes in 31 OSCC samples. (**A**) The mRNA expression pattern of 11 drug response-linked genes. The total mRNA was isolated from fresh tumor tissues and were detected using custom PCR array following the manufacturer’s instructions. The clustergram results of three independent experiments were analyzed using the SA Biosciences online tool. (**B**) Venn diagram showed the gene expression pattern of drug resistance genes in tumor samples. Samples 9 and 10 overexpress most of the drug response-linked genes.

We analyzed the expression profiles of certain MDR-linked genes by PCR array to understand the sensitivity of cancer types to anticancer treatments. In 2009 Yukio *et al*., identified five novel genes which possess great potential for predicting the efficacy of cisplatin-based chemotherapy against OSCC using Affymetrix U133 Plus 2.0 microarray^22^. The analysis of the mRNA expression pattern of tumor-specific genes has been employed to understand the association with risk habits and the clinicopathological profile of OSCC cases in Indian population^23^. In this study, we observed the list of drug resistance linked genes was highly expressed in well-differentiated tumor samples. Moreover, the analysis showed that there was a clear overexpression of resistance genes in tumors as compared to normal tissue (Fig. 1A). Prior to gene expression analysis, patients’ samples were analyzed and categorized based on their clinical stage. It was observed that samples 1, 2, 7, 9, 10, 11, 14, 17, 19, 24 and 27 were found to be well differentiated which mostly resembles normal tissue architecture and usually has a good prognosis; samples 5, 6, 16, 18, 20, 23, 26, 28 and 31 were moderately differentiated i.e. an intermediate forms of tumor with either good or bad prognosis. Samples 3, 4, 8, 12, 13, 15, 21, 22, 25, 29 and 30 were found to be poorly differentiated which metastasis easier and their prognosis will generally be worser^24^ (Supplementary Fig. 4).

Interestingly, it has been noticed that the expression pattern of genes linked to drug response is well correlated with the clinical and pathological characteristics of the patients (Supplementary Table 1 and Fig. 1B). We observed that the average drug response-linked genes expression was higher in the studied male (average RQ = 2.24) population than the female population (average RQ = 1.50). This has been reflected in the drug response that the average apoptotic priming of female (57.15%) was higher than the average apoptotic priming of male (52.22%). The correlation between the drug response linked gene expression with the % apoptotic priming was higher in female tumor samples (−0.532) when compared to male tumor samples (−0.7222) and the differences in drug response between the gender were found to be 19% (Supplementary Table 2).

Similarly, Gillet *et al*.^25^ earlier employed the gene expression pattern to reveal clinical anticancer drug resistance^25^. Membrane drug efflux transporters such as ABCB1, ABCG2 and ABCC1 were generally overexpressed in the patient’s tumor samples according to all clinical and pathological criteria^26^. Many of these ABC transporters have been shown to efflux out most of the anticancer drugs that were employed in this study^27–29^. Recently, a genome-wide analysis study illustrated the association between variation in the ABCB1 and ABCB4 gene regions and the risk of gallbladder cancer in Indian population^30^. The expression of transporter genes such as ABCB1, ABCG2 and ABCC1 was found to be increased in the studied Indian OSCC tissue samples (3, 4, 5, 7, 9, 10, 14, 16, 17, 18, 19, 20, 23 and 31), which were found to be highly progressive histologically (Fig. 1A and Supplementary Table 1). Overexpression of ABC transporters such as P-gp, MRP and BCRP has been shown to be responsible for the major portion of MDR^31–33^ and therefore using the expression pattern of ABC transporters for predicting tumor response will be useful information before therapy.

The cyclin-dependent kinases promote cell cycle arrest in response to many stimuli. Alteration in cell cycle regulators significantly renders chemotherapeutic drugs ineffective^34^. In this study, we found that the cell cycle regulators such as CDKN1A and CDK2 were found to be overexpressed in tumor samples 1, 2, 3, 4, 5, 9, 10, 11, 14, 18, 20, 21, 23, 27 and 31 (Supplementary Table 1). High STAT5 levels mediate imatinib resistance and indicate disease progression in chronic myeloid leukemia^35^. Multidrug-resistant cancer cells frequently overexpress the 110-kD LRP protein. LRP overexpression has been found to predict a poor response to chemotherapy in acute myeloid leukemia and ovarian carcinoma^36^. In this analysis, the tumor progression protein LRP1 and cytokine-inducible transcription factor STAT5B were found to be overexpressed in samples 1, 2, 3, 5, 9, 10, 11, 14, 18, 20 and 24 (Supplementary Table 1). Mutations in p53 protein and loss of TP53 function confer MDR in several breast cancer tumor sub-types^31,37,38^. We observed that TP53 was downregulated in tumor samples 1, 2, 4–8, 10–12, 14–21, and 23–31 (Supplementary Table 1).

**Figure 2:**
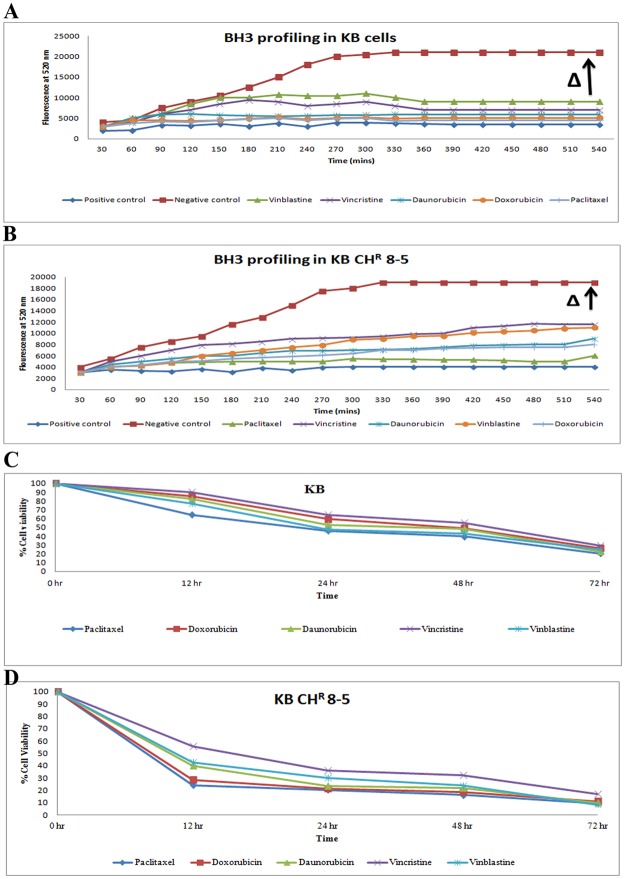
(**A**,**B**) The % apoptotic priming in parental KB and drug-resistant KB CHR 8 5 cells. Cells were exposed to different anticancer drugs at equal concentrations (1 μM) for the different time period. The % apoptotic priming was confirmed by treating the cells with DMSO (positive control) and FCCP (negative control), respectively. It was observed that paclitaxel showed maximum apoptotic priming when compared to the other anticancer drugs (Fig. S6). (**C**,**D**) Cytotoxicity of chemotherapeutic drugs (1 µM) on parental KB and drug-resistant KB CHR 8–5 cell lines. Time-dependent curves of chemotherapeutic drugs at fixed concentrations in parental KB and resistant KBCHR 8–5 cell lines. The values shown were the average of experiments each done in triplicate.

The range of gene expression could determine the sensitivity of drugs to the tumor samples. The range of Ct values of ABCB1 for all 31 tumors was between 23 and 31. Those tumors exhibiting Ct values from 19 to 23, the first quartile, may show resistance to anticancer therapy; tumors which showed Ct values from 23 to 26, the second quartile, may be moderately sensitive to therapy; whereas, the third quartile (Ct values 26 to 31) and fourth quartile (Ct values 31 to 32) were tumors with low expression levels of ABCB1 that could be sensitive to anticancer treatment. Similarly, drug response could be easily predicted based on the pattern of expression of other drug response genes (Supplementary Fig. 5). The gene expression pattern alone was not found to be adequate for clinical correlation of drugs due to post-translational modifications of expressed genes and poor dynamic range^39^. Hence, to further confirm tumor sensitivity to chemotherapeutic drugs, we carried out BH3 profiling in primary cells isolated from the tumor samples. As a preliminary study, before doing BH3 profiling in tumor samples, we determined the expression pattern for genes related to drug response in two different cancer cell lines, including oral tumor-derived KB cell lines and its drug-resistant sub-type KB CH^R^ 8–5. We observed that paclitaxel showed higher apoptotic priming in drug-sensitive parental KB cells when compared to other chemotherapeutic drugs (Fig. 2A). Similarly, significant % apoptotic priming was induced in the case of parental KB oral cancer cell lines when compared to their drug-resistant sub-type KB CH^R^ 8–5. It has been found that paclitaxel induces significant apoptotic priming in these drug-resistant KB CH^R^ 8–5 cells, whereas vincristine showed very poor apoptotic priming (Fig. 2B) in the KB CH^R^ 8–5 cells. Thus, paclitaxel may be considered the most effective cytostatic drug against the drug-resistant KB CH^R^ 8–5 cells than all other drugs studied in this investigation. This pattern of drug sensitivity was also reflected in an MTT-based cytotoxicity assay (Fig. 2C,D). This further validates the importance of understanding apoptotic priming by BH3 profiling when predicting the drug response of tumor cells.

**Figure 3:**
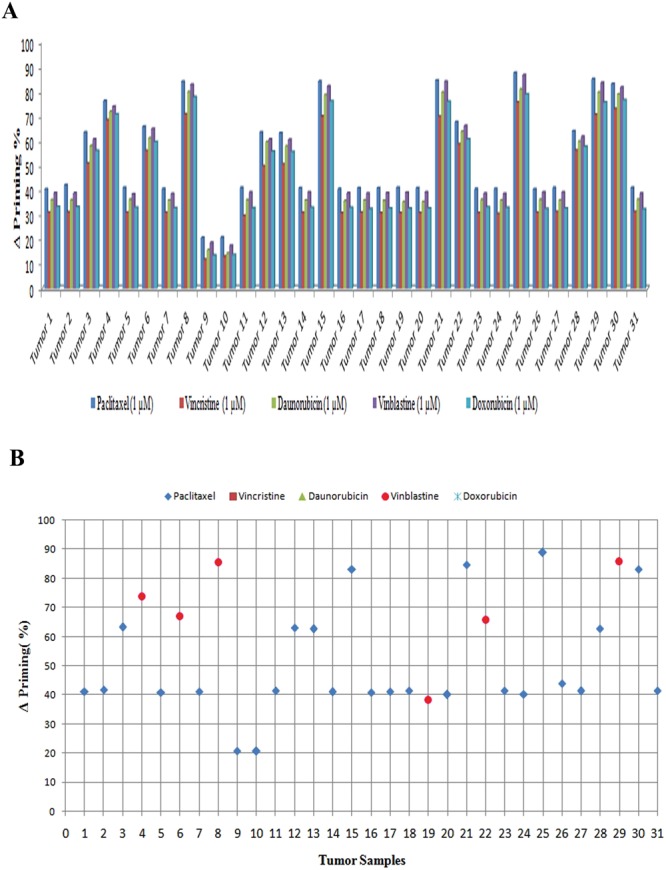
Analysis of apoptotic priming in OSCC samples by BH3 (Bcl2 Homology-3) profiling. (**A**) Primary cells isolated from tumor biopsies were treated with different chemotherapeutic drugs and BH3 profiling was performed. Individual BH3 profiling analysis was performed using triplicates for controls and BIM (BCL-2-interacting mediator of cell death) BH3 peptide, and the expressed values were the average of three different readings. (**B**) Drug of choice for the tumor samples based on % apoptotic priming. The maximum priming of a drug against each tumor was identified and differences with the priming efficacy of other drugs were plotted.

**Figure 4:**
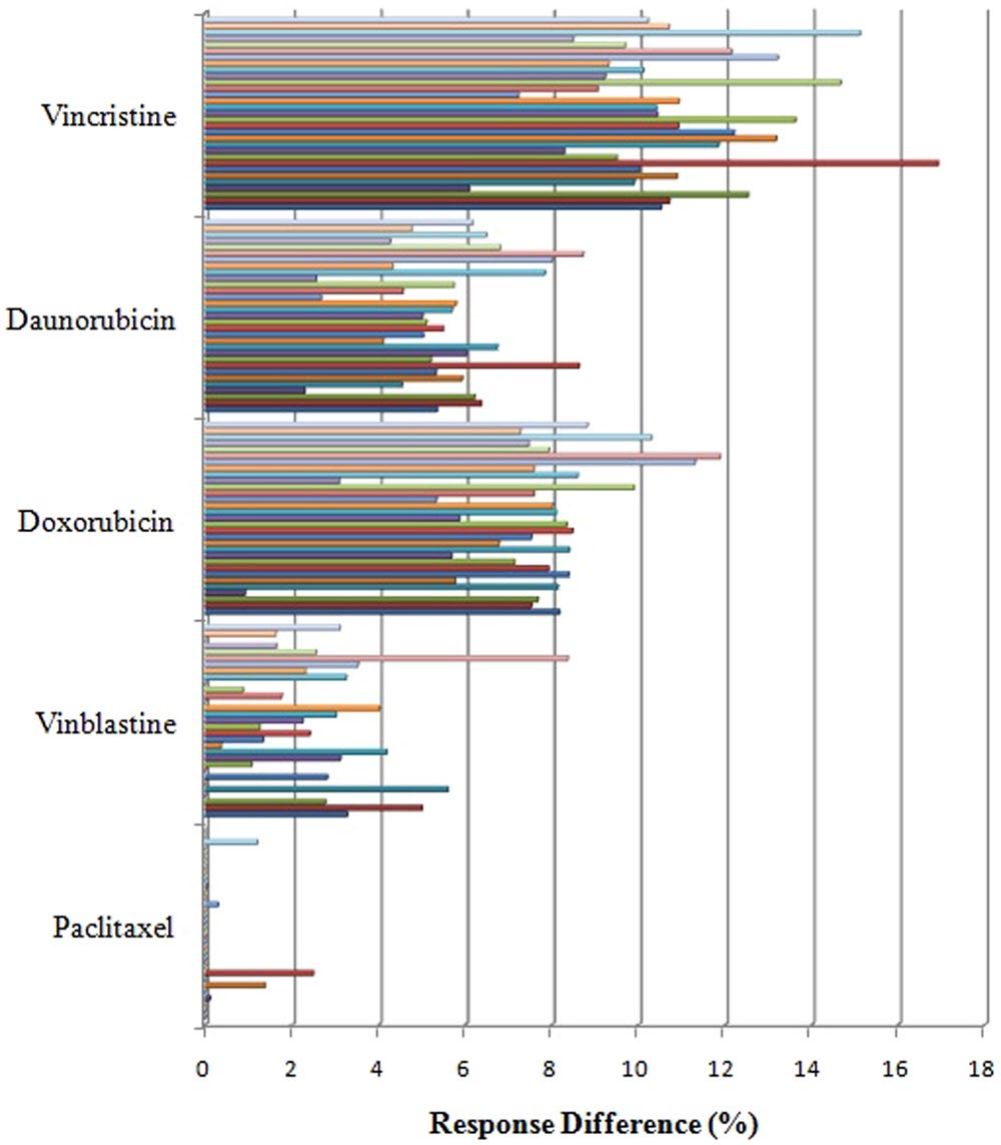
Best priming drug and response differences between drugs. Response Difference (RD) between the best performing drug (BPD) and the other drugs were observed as per Equations (1 and 2).

**Figure 5:**
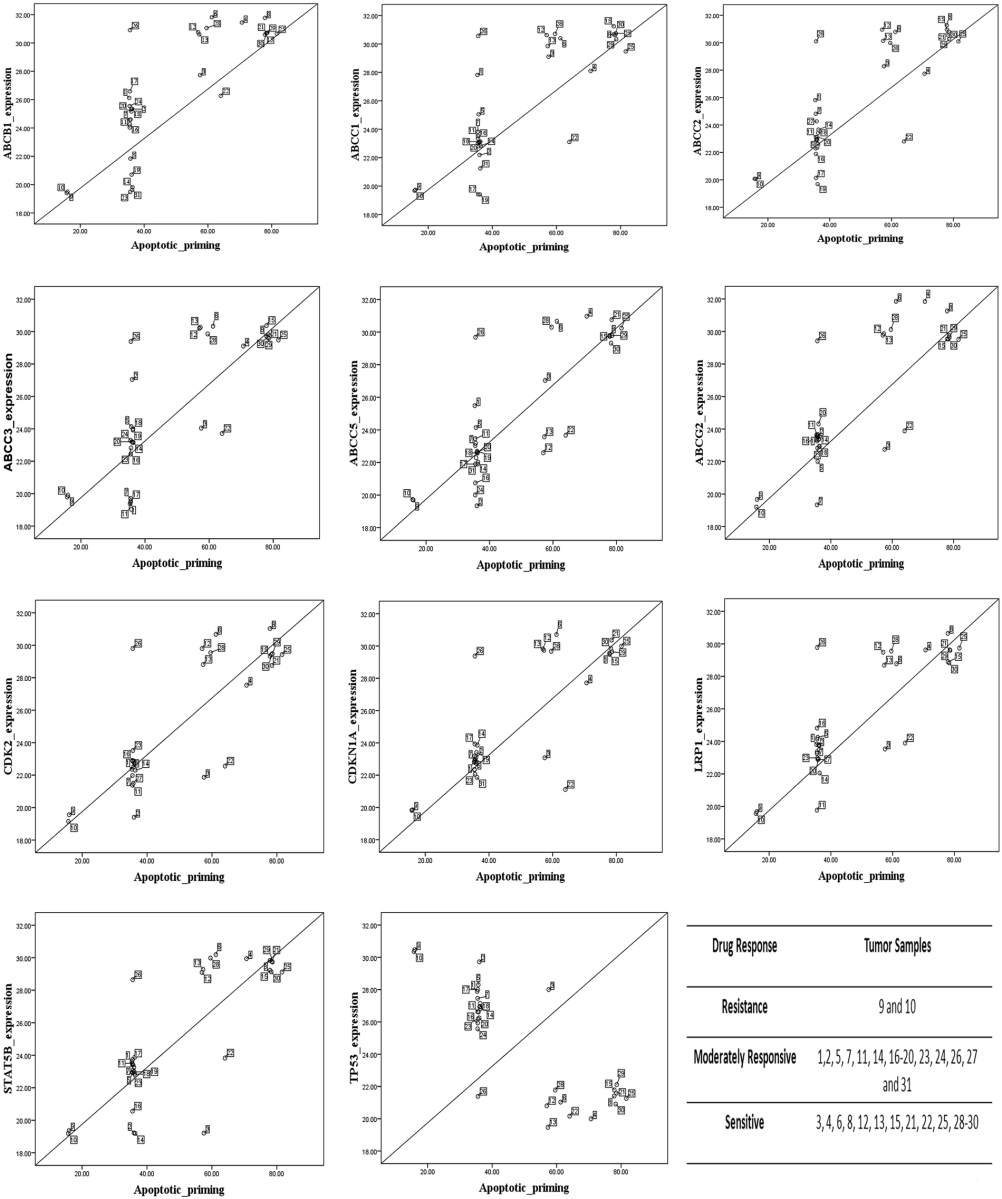
Pearson correlation of expression pattern of 11 MDR-linked genes with the % apoptotic priming induced by chemotherapeutic drugs. The Statistical Package, IBM SPSS (Version 21), and Microsoft Excel 2007 (Roselle, IL) were used for the statistical and graphical evaluations. Tumors were classified as resistant, moderately responsive and sensitive based on their response to the chemotherapeutic drugs.

We observed significant apoptotic priming in the patient’s tumor samples to chemotherapeutic drugs depending upon the type of gene expression pattern concerning genes related to drug response. As expected, we found that up-regulation of MDR-linked genes facilitated the survival of all tumor samples. Tumor samples 9 and 10, which had the highest level of expression of drug resistance genes, were found to be resistant to all the chemotherapeutic drugs studied when compared to other tumor samples. Furthermore, tumor samples which expressed at least one of the drug resistance markers (viz. 1, 2, 5–7, 11, 14, 16–20, 23, 24, 27 and 31) were also found to be resistant to anticancer therapy. The tumor samples which were generally not showing any drug resistance gene expression (viz. 4, 8, 15, 21, 25, 26, 28, 29 and 30) were found to be sensitive to chemotherapeutic drugs. The mitochondrial apoptotic priming during drug treatment in tumor samples 3, 6, 12, 13 and 22 were found to be only partial (Fig. 3A). It should be noted that the MDR gene expression pattern probably reflects the biologic state of the OSCC since the patients who were the source of the analyzed tumor samples were not treated with any chemotherapeutic agents. Among all the drugs studied, paclitaxel-induced 40–45% apoptotic priming; vinblastine and daunorubicin induced 35–40%; and doxorubicin and vincristine-induced 30–35% apoptotic priming in the tumor cells. The maximum apoptotic priming was observed by paclitaxel (90%) in tumor sample 25 (Supplementary Fig. 6). Equation (1) was used to check the apoptotic priming potential of drugs *j* (1 to 5) in the tumor samples *i* (1 to 31). Figure 3B shows the drug of choice for each tumor sample on the basis of apoptotic priming. We noticed that paclitaxel was the best priming drug (BPD), effective in 25 tumors out of 31 samples, followed by vinblastine, effective in 6 tumors out of 31 samples. RD values for 31 tumors were calculated using Equation (2) as per the Supplementary Scheme 1. We found that paclitaxel showed highest apoptotic priming %, the average RD was found to be very less (0.177) i.e. negligible and hence through BPD and RD, it was proved that paclitaxel was the best priming drug. As per Equations (1 and 2), the average RD of the second-ranking drug (vinblastine) with paclitaxel was 2.3% in these 25 tumor samples. The RD of paclitaxel with vinblastine in these 6 tumors was only 0.77%, which could not be considered significant. Daunorubicin was ranked next in efficacy with RD of 5.54% from paclitaxel. We observed that there was a larger RD between paclitaxel and doxorubicin (7.61%) and vincristine (10.9%) in the tumor samples (Fig. 4). The average RD computation of paclitaxel was found to be less than 1. Furthermore, we noticed that about 80% of tumors showed a good response to the drug paclitaxel (Fig. 3B). Hence, it is evident that paclitaxel should be considered as the most potentially effective drug for the studied cohort population from among the other drugs studied. However, it is not rational to conclude that the other three drugs studied (i.e. daunarubicin, doxorubicin and vincristine) were ineffective treatments for the OSCC samples; rather their efficacy was less when compared to the performance of paclitaxel. To further validate these findings, we carried out a correlation analysis between MDR-linked gene expression and percentage of apoptotic priming (average priming values of all five drugs) in order to help design better chemotherapy options for oral carcinoma. On the basis of correlations between expression patterns of genes related to drug response and apoptotic priming, it can be possible to classify the tumors as sensitive (3 < 13 < 12 < 28 < 6 < 22 < 4 < 30 < 15 < 8 < 21 < 29 < 25), moderately responsive (19 < 24 ≤ 20,16,14,7,11,18,27,31 ≤ 23 ≤ 5,1,17,2 < 26) and resistant (9 and 10) to anticancer therapy (Fig. 5). To further validate this classification we employed Partitioning Around Medoids (PAM) and the results were cluster plotted. The PAM is a machine learning algorithm which partitions the dataset into clusters^40^. The Ct-values of drug response linked gene expression and % apoptotic priming were analyzed by the PAM algorithm and the tumors were clustered as sensitive, moderately responsive and resistant (Supplementary Fig. 7). Therefore, the present results could be used to assign the drug responses for any *in vitro* cell lines and even could be translated to the cancer clinics to predict the drug response before therapy.

**Figure 6:**
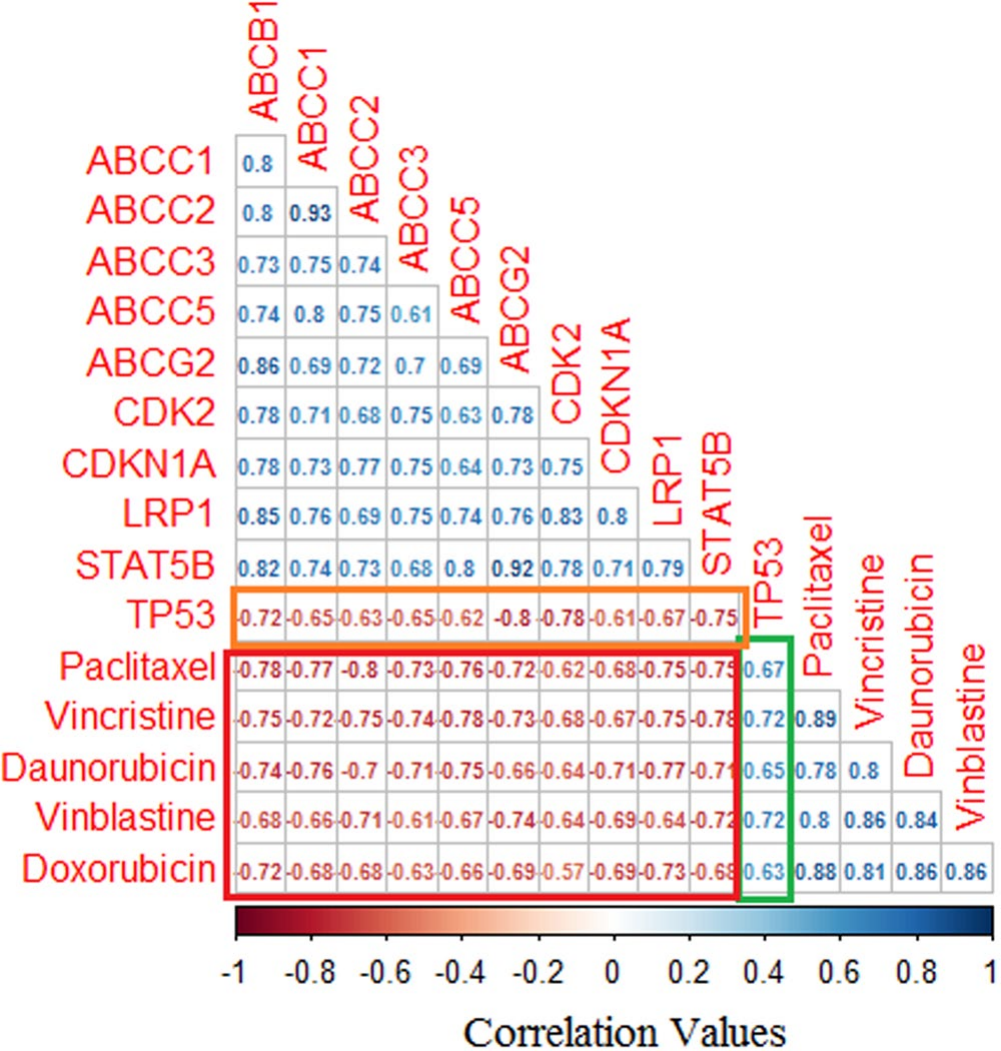
Correlation between MDR-linked gene expression and the efficacy of each anticancer drug. All the MDR-linked genes employed in this study were negatively correlated with chemotherapeutic drug performance, whereas TP53 expression was positively correlated with the anticancer drug performance.

To the best of our knowledge, this is the first time OSCC tumors have been classified based on their gene expression pattern and apoptotic priming status. When TP53 was overexpressed, all the drugs tested were found to be effective (green box in Fig. 6). The negative correlation of TP53 with other genes (orange box in Fig. 6) inferred that TP53 suppressed the overexpression of other MDR-linked genes in the tumor samples. Furthermore, the expression of all the MDR-linked genes was negatively correlated with anticancer drug performance (red box in Fig. 6). The highest negative correlation (between paclitaxel performance and MDR-linked gene expression) further confirmed paclitaxel as the best choice of treatment for the studied OSCC patients. Hence, we plotted the percentage of apoptotic priming of paclitaxel alone with the expression of each drug response linked genes to show linear regression^41,42^ (brown lines; Supplementary Fig. 8) and to reveal locally weighted polynomial regression (blue dashed lines in Supplementary Fig. 8). The lines indicate that there was no perfect linear relationship observed. The uncertainty in this relationship suggests that the correlation between MDR gene expression and apoptotic priming might also depend on other factors such as age, sex, tumor grade and tumor stage.

The linear prediction has previously been employed to identify the most effective chemotherapy drug based on MDR-linked gene expression data^43,44^. Recently Baidehi *et al*., (2017) have revealed a set of differentially methylated DNA regions which were replicated in 60–90% cohort of Indian OSCC patients^45^. In this study, we observed low R-Square value (0.3331) and high p-value (0.5875), which questions the reliability of this linear prediction model. The confidence level of the drug of choice prediction based on 31 tumor samples was only 33%. Therefore, to confirm the drug of choice for this OSCC population the number of tumor samples may be increased so that the personalized choice of drug for oral cancer patients could be more accurately predicted. Previously, there were number of studies illustrating drug prediction based on the small sample number. Montero *et al*.^14^ employed 24 bone marrow primary chronic myeloid leukemia samples to predict imatinib response and 16 ovarian adenocarcinoma samples to predict carboplatin response using BH3 profiling^14^. Burger *et al*.^46^ analyzed the mRNA expression levels of BCRP, LRP, MRP1, MRP2 and MDR1 based on 59 breast tumor samples^46^. Li Su *et al*.^47^ examined the association between the MDR1 gene of gastrointestinal tumors and resistance to chemotherapeutic drugs using 38 colon cancer samples, 46 esophageal cancer samples and 42 gastric cancer samples^47^. Though, the number of samples in this study was only 31, the present findings can be applicable to OSCC when we further account other clinical features like age, sex, tumor grade, tumor stage and clinical staging of the patients. Analyzing these multiple clinical and experimental features using statistical methods like multilinear regression (MLR), MLR-Log and Least Square Method (LSM) will precisely predict the drug response before therapy.

## Conclusions

According to the present findings, correlation of MDR-linked gene expression pattern with the results of BH3 apoptotic profiling can be a potential predictive strategy to identify the best drug option among many therapeutic drugs available for OSCC patients. It is important to note that we used tissue samples collected prior to any treatment; thus, reveals the biological status of tumors which will assist in the selection of drug of choice to a patient in a personalized manner.

## Electronic supplementary material

Supplementary Files
