# Supplementary material for: Predicting Tumor Sensitivity to Chemotherapeutic Drugs in Oral Squamous Cell Carcinoma Patients
Source: Sci Rep. 2018 Oct 19;8:15545. doi: 10.1038/s41598-018-33998-4 (PMC6195614; doi:10.1038/s41598-018-33998-4)
Supplement: Supplementary file 1 — Supplementary Files [file 41598_2018_33998_MOESM1_ESM.docx]

**Predicting Tumor Sensitivity to Chemotherapeutic Drugs in**

**Oral Squamous Cell Carcinoma Patients**

**Beaulah Mary Robert ^1^, Muralidharan Dakshinamoorthy ^2^, Ganapathyagraharam Ramamoorthy Brindha ^2^,**

**Muthu Dhandapani ^3^, Rathiga Thangaiyan ^1^, Ganesan Muthusamy ^1^, R. Madhavan Nirmal ^4^, Nagarajan Rajendra Prasad *^,1^**

**Corresponding Author: drprasadnr@gmail.com**

**Supplementary Figure 1**

mRNA isolation

Correlation of gene expression analysis and cell based apoptotic assays

Drug response prediction

Personalized choice of therapy

Baseline drug

Response Gene

Expression

analysis


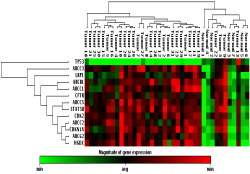

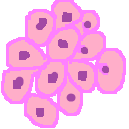


Single suspension

primary cells


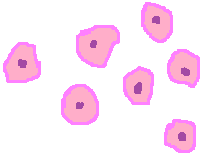


Tissue biopsies

in RNA later


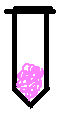


Tissue biopsies

in cell culture

medium


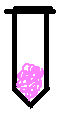


Tumor biopsy

BH3 profiling analysis


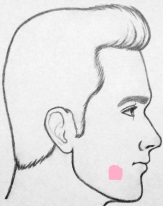


**A**

**B**


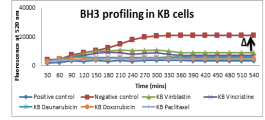


Apoptotic analysis BH3 profiling analysis

by BH3 profiling

Cytotoxicity of Cytotoxicity analysis

anticancer drugs

Drug response

Experimental Cell lines

KB & KBCH^R^ 8-5

Cell based assays


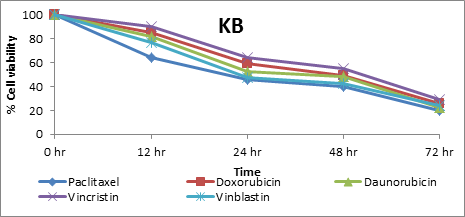


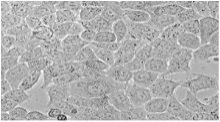

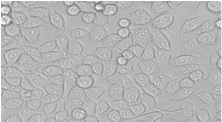


**Supplementary Figure 2**

**MASS SPECTrometry Report**

Product: Peptide

[M+3H]3+

[M+2H]2+

Date and Time: 2016-3-8 10:06:41

User : R.S

Sample : ER-20

MW : 2309.61

Lot No. : ER-20/503115

Probe: ESI Probe bias: +4.5kv

Nebulizer Gas Flow: 1.5L/min Detector: 1.5kv

CDL: -20.0v T. Flow: 0.2ml/min

CDL Temp: 250℃ B. conc: 50%H2O/50%ACN

Block Temp: 200℃

**Supplementary Figure 3**

**Supplementary Figure 4**


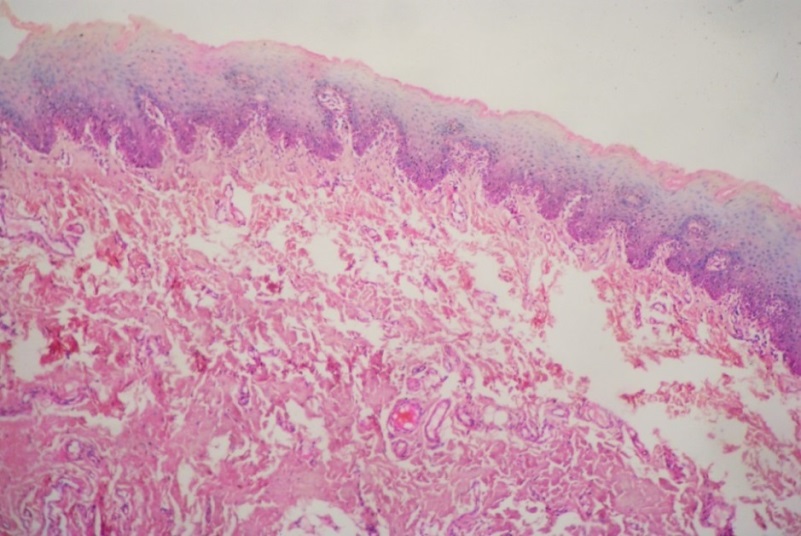

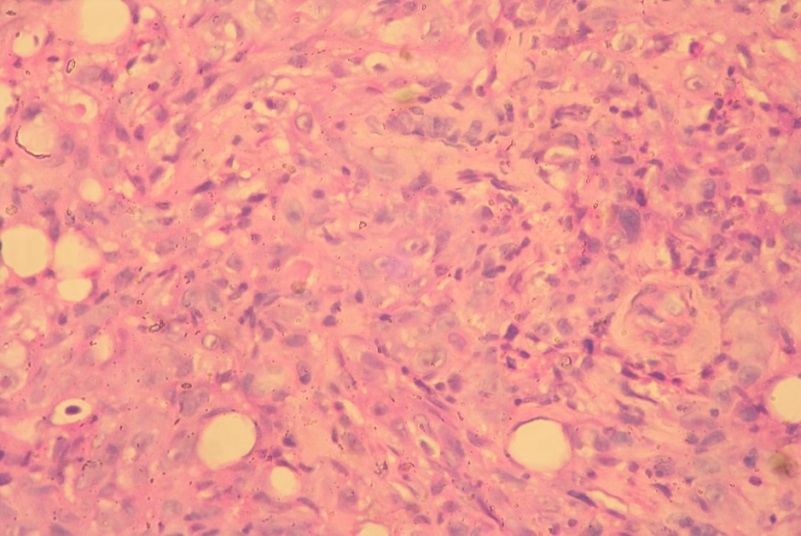

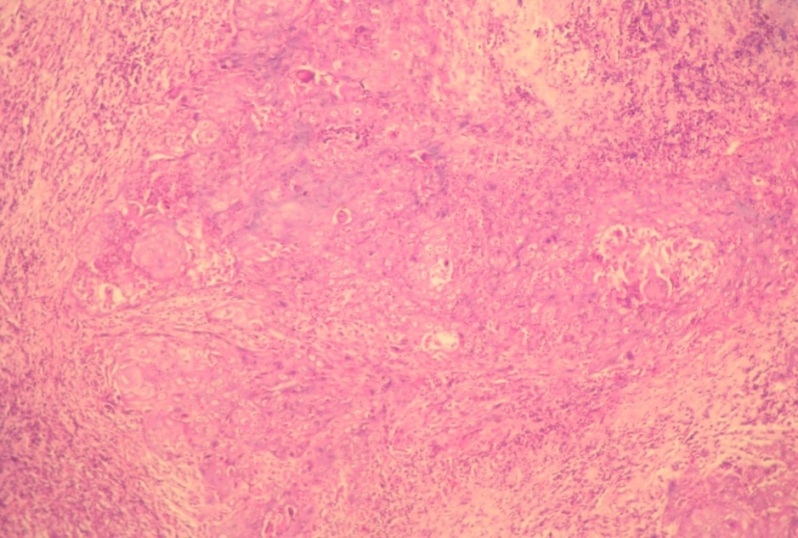

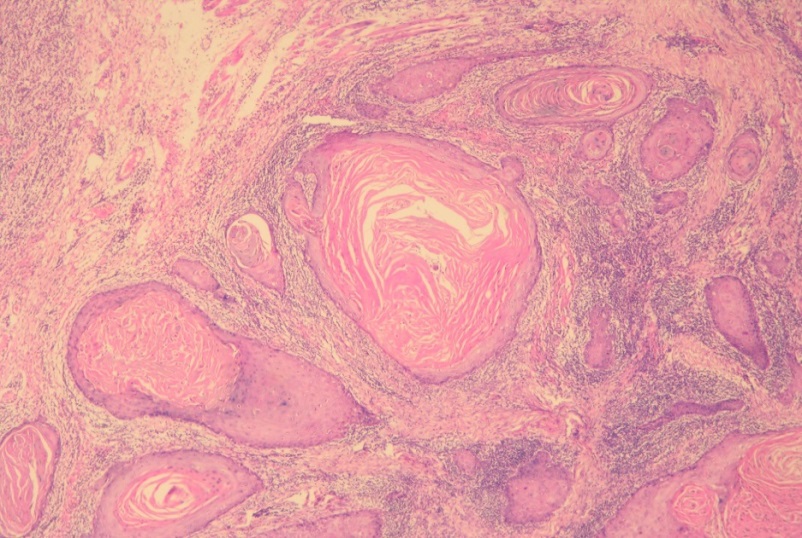


**Sample no. 1 - Normal**

**Sample no. 21 - Poorly differentiated**

**Sample no. 5 - Moderately differentiated**

**Sample no. 9 - Well differentiated**

**Supplementary Figure 5**

**
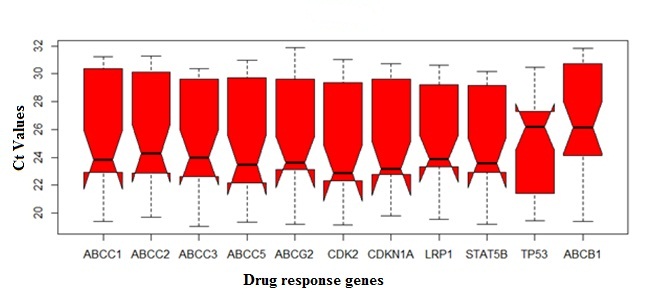
**

**
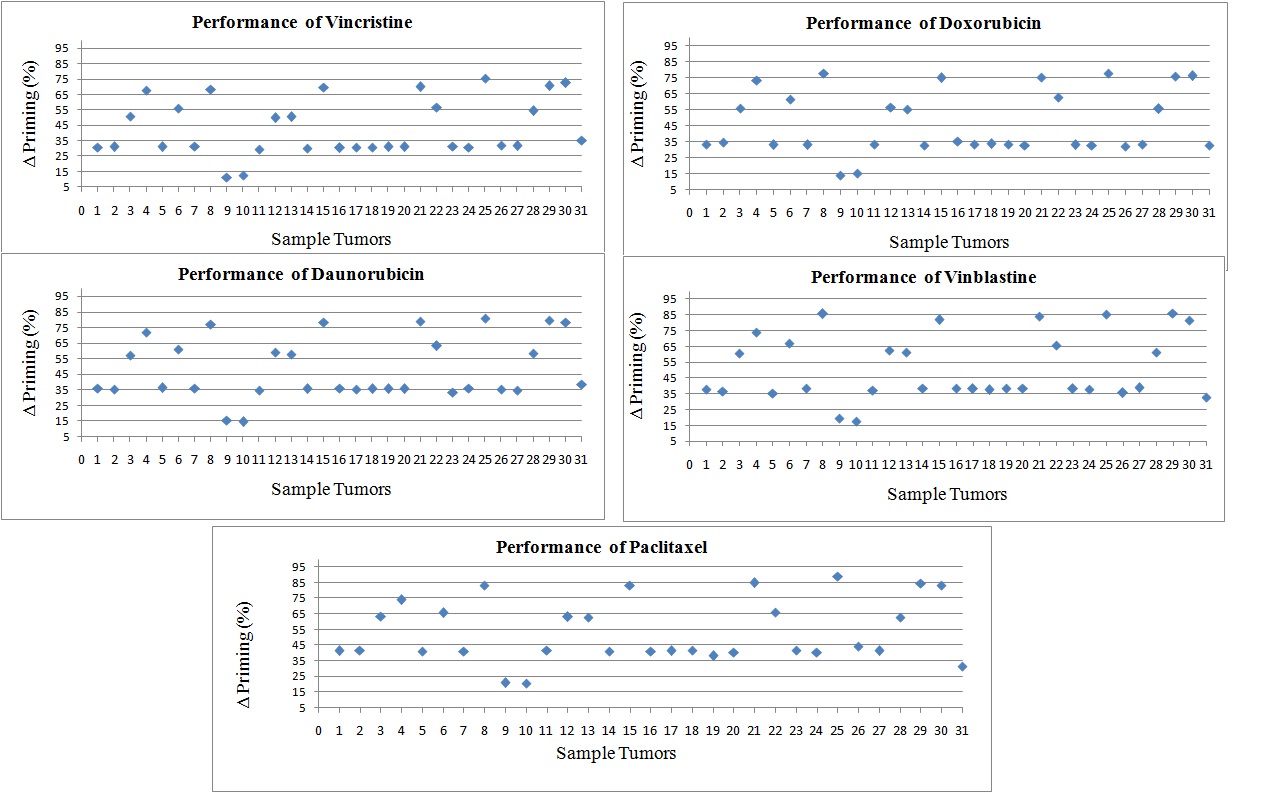
Supplementary Figure 6**

**Supplementary Figure 7**

**
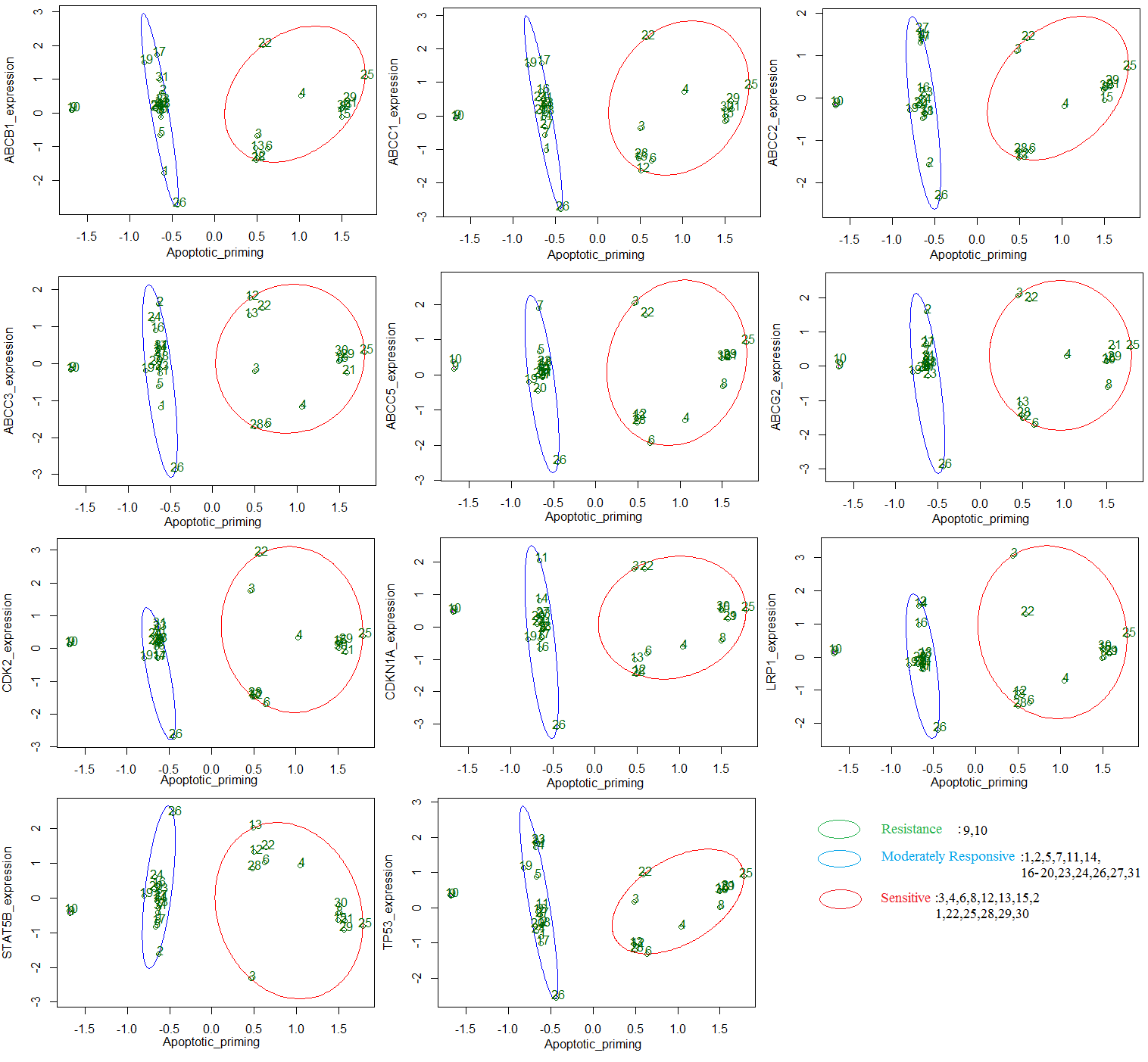
**

**Supplementary Figure 8**

**
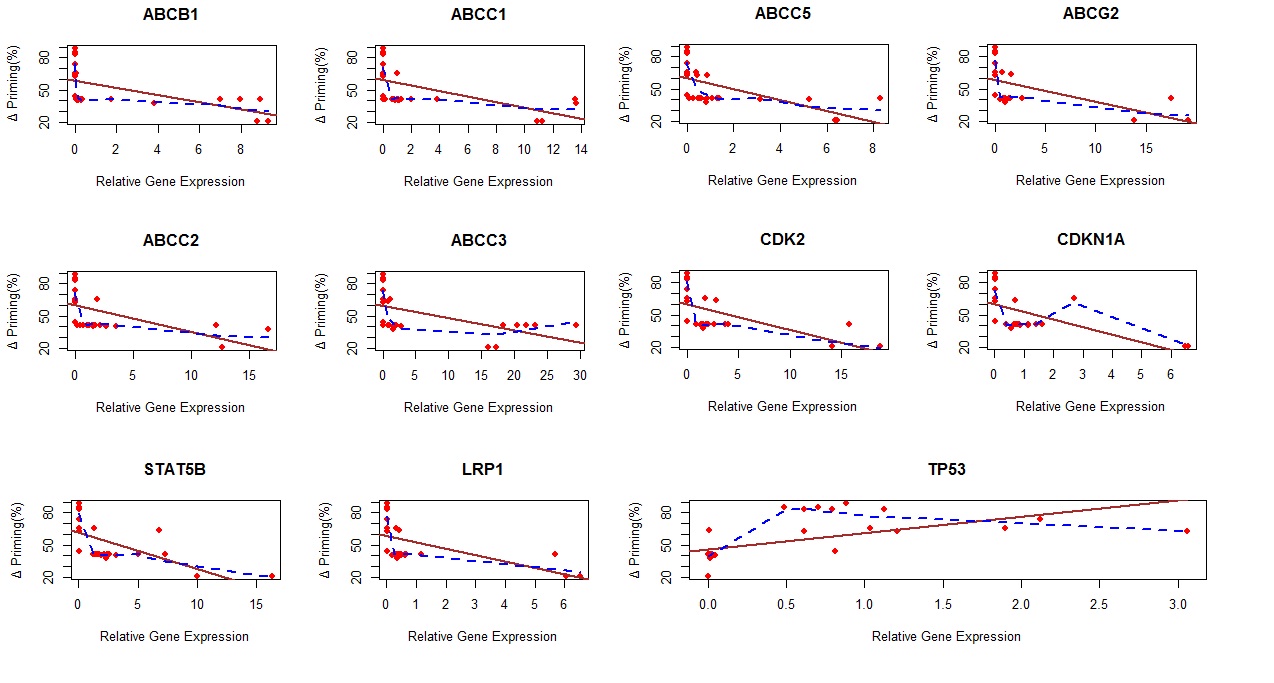
**

| S.No.  **Supplementary Table 1.** Multidrug drug resistance genes expression in oral tumor tissue samples | Groups | | Total (*N*=31) | **ABCB1** | | **ABCG2** | | **ABCC1** | |
| --- | --- | --- | --- | --- | --- | --- | --- | --- | --- |
|  |  |  |  | Expression | Overexpression | Expression | Overexpression | Expression | Overexpression |
|  | **Sex** | M | 22 | 19 (86.4%) | 3 (13.6%) | 21 (95.4%) | 1 (4.6%) | 18 (81.8%) | 4 (18.2%) |
|  |  | F | 9 | 8 (88.9%) | 1 (11.1%) | 7 (77.8%) | 2 (22.2%) | 8 (88.9%) | 1 (11.1%) |
| **Average** | | |  | **27(87.09%)** | **4 (12.9%)** | **28(90.32%)** | **3 (9.67%)** | **26(83.87%)** | **5 (16.12%)** |
|  | **Age** | 35-45 | 5 | 5 (100%) | 0 | 4 (80%) | 1 (20%) | 5 (100%) | 0 |
|  |  | 45-55 | 14 | 10 (71.4%) | 4 (28.6%) | 13 (92.9%) | 1 (7.1%) | 11 (78.6%) | 3 (21.4%) |
|  |  | 55-65 | 9 | 9 (100%) | 0 | 8 (88.9%) | 1 (11.1%) | 7 (77.8%) | 2 (22.2%) |
|  |  | 65-75 | 3 | 3 (100%) | 0 | 3 (100%) | 0 | 3 (100%) | 0 |
| **Average** | | |  | **27(87.09%)** | **4 (12.90%)** | **28(90.32%)** | **3 (9.67%)** | **26(83.87%)** | **5 (16.12%)** |
|  | **Tumor Grade** | Well differentiated | 9 | 8 (88.9%) | 1 (11.1%) | 8 (88.9%) | 1(11.1%) | 7 (77.8%) | 2 (22.2%) |
|  |  | Moderately differentiated | 11 | 8 (72.7%) | 3 (27.3%) | 9 (81.8%) | 2 (18.2%) | 8 (72.7%) | 3 (27.3%) |
|  |  | Poorly differentiated | 11 | 11 (100%) | 0 | 11 (100%) | 0 | 9 (81.8%) | 2 (18.2%) |
| **Average** | | |  | **27(87.09%)** | **4 (12.90%)** | **28(90.32%)** | **3 (9.67%)** | **26(83.87%)** | **5 (16.12%)** |
|  | **Tumor Stage** | II | 2 | 1 (50%) | 1 (50%) | 2 (100%) | 0 | 2 (100%) | 0 |
|  |  | III | 3 | 2 (66.7%) | 1 (33.3%) | 2 (66.7%) | 1 (33.3%) | 2 (66.7%) | 1 (33.3%) |
|  |  | IV | 3 | 3 (100%) | 0 | 3 (100%) | 0 | 3 (100%) | 0 |
|  |  | Unknown | 23 | 21 (91.3%) | 2 (8.7%) | 21 (91.3%) | 2 (8.7%) | 19 (82.6%) | 4 (17.4%)s |
| **Average** | | |  | **27(87.09%)** | **4 (12.90%)** | **28(90.32%)** | **3 (9.67%)** | **26(83.87%)** | **5 (16.12%)** |
|  | **Clinical staging** | Alveolus | 9 | 8 (88.9%) | 1 (11.1%) | 8 (88.9%) | 1 (11.1%) | 8 (88.9%) | 1 (11.1%) |
|  |  | Buccal | 8 | 8 (88.9%) | 0 | 8 (88.9%) | 0 | 7 (87.5%) | 1 (12.5%) |
|  |  | Gingiva | 7 | 6 (85.7%) | 1 (14.3%) | 5 (71.4%) | 2 (28.6%) | 6 (85.7%) | 1 (14.3%) |
|  |  | Alveolus & Buccal | 7 | 5 (71.4%) | 2 (28.6%) | 7 (87.5%) | 0 | 5 (71.4%) | 2 (28.6%) |
| **Average** | | |  | **27(87.09%)** | **4 (12.9%)** | **28(90.32%)** | **3 (9.67%)** | **26(83.87%)** | **5 (16.12%)** |

| S.No. | Groups | | Total (*N*=31) | **ABCC2** | | **ABCC3** | | **ABCC5** | |
| --- | --- | --- | --- | --- | --- | --- | --- | --- | --- |
|  |  |  |  | Expression | Overexpression | Expression | Overexpression | Expression | Overexpression |
|  | **Sex** | M | 22 | 15(68.18%) | 4 (18.18%) | 17(77.27%) | 4 (18.18%) | 18 (81.81%) | 3 (13.63%) |
|  |  | F | 9 | 9 (100 %) | 0 | 7 (77.78%) | 2 (22.22%) | 7 (77.78%) | 2 (22.22%) |
| **Average** | | |  | **24(77.42%)** | **4 (12.9%)** | **24(77.42%)** | **6 (19.35%)** | **25 (80.65%)** | **5 (16.13%)** |
|  | **Age** | 35-45 | 5 | 5 (100%) | 0 | 4 (80%) | 1 (20%) | 4 (80%) | 1 (20%) |
|  |  | 45-55 | 14 | 11(78.57%) | 1 (7.14%) | 11(78.57%) | 3 (21.43%) | 12 (85.71%) | 1 (7.14%) |
|  |  | 55-65 | 9 | 6 (66.67%) | 3 (33.33%) | 8 (88.89%) | 1 (11.11%) | 6 (66.67%) | 3 (33.33%) |
|  |  | 65-75 | 3 | 2 (66.67%) | 0 | 1 (33.33%) | 1 (33.33%) | 3 (100%) | 0 |
| **Average** | | |  | **24(77.42%)** | **4 (12.9%)** | **24(77.42%)** | **6 (19.35%)** | **25 (80.65%)** | **5 (16.13%)** |
|  | **Tumor Grade** | Well differentiated | 9 | 7 (77.78%) | 1 (11.11%) | 6 (66.67%) | 2 (22.22%) | 7 (77.78%) | 2 (22.22%) |
|  |  | Moderately differentiated | 11 | 8 (72.73%) | 2 (18.18%) | 8 (72.73%) | 3 (27.27%) | 8 (72.73%) | 2 (18.18%) |
|  |  | Poorly differentiated | 11 | 9 (81.82%) | 1 (9.09%) | 10(90.90%) | 1 (9.1%) | 10 (90.90%) | 1 (9.1%) |
| **Average** | | |  | **24(77.42%)** | **4 (12.9%)** | **24(77.42%)** | **6 (19.35%)** | **25 (80.65%)** | **5 (16.13%)** |
|  | **Tumor Stage** | II | 2 | 2 (100%) | 0 | 2 (100%) | 0 | 2 (100%) | 0 |
|  |  | III | 3 | 2 (66.67%) | 1 (33.33%) | 1 (33.33%) | 2 (66.67%) | 2 (66.67%) | 1 (33.33%) |
|  |  | IV | 3 | 2 (66.67%) | 0 | 3 (100%) | 0 | 2 (66.67%) | 1 (33.33%) |
|  |  | Unknown | 23 | 18(78.26%) | 3 (13.04%) | 18(78.26%) | 4 (17.39%) | 19 (82.61%) | 3 (13.04%) |
| **Average** | | |  | **24(77.42%)** | **4 (12.9%)** | **24(77.42%)** | **6 (19.35%)** | **25 (80.65%)** | **5 (16.13%)** |
|  | **Clinical staging** | Alveolus | 9 | 6 (66.67%) | 1 (11.11%) | 7 (77.78%) | 1 (11.11%) | 7 (77.78%) | 1 (11.11%) |
|  |  | Buccal | 8 | 5 (62.5%) | 2 (25%) | 6 (75%) | 2 (25%) | 5 (62.5%) | 3 (37.5%) |
|  |  | Gingiva | 7 | 6 (85.7%) | 1 (14.3%) | 5 (71.4%) | 2 (28.6%) | 6 (85.7%) | 1 (14.3%) |
|  |  | Alveolus & Buccal | 7 | 7 (100%) | 0 | 6 (85.7%) | 1 (14.3%) | 7 (100%) | 0 |
| **Average** | | |  | **24(77.42%)** | **4 (12.9%)** | **24(77.42%)** | **6 (19.35%)** | **25 (80.65%)** | **5 (16.13%)** |

| S.No. | Groups | | Total (*N*=31) | **CDKN1A** | | **CDK2** | | **TP53** | |
| --- | --- | --- | --- | --- | --- | --- | --- | --- | --- |
|  |  |  |  | Expression | Overexpression | Expression | Overexpression | Expression | Downexpression |
|  | **Sex** | M | 22 | 14(63.64%) | 5 (22.73%) | 19 (86.4%) | 2 (9.1%) | 5 (22.73%) | 17 (77.27%) |
|  |  | F | 9 | 6 (66.67%) | 1 (11.11%) | 8 (88.89%) | 1 (11.11%) | 1 (11.11%) | 8 (88.89%) |
| **Average** | | |  | **20(64.52%)** | **6 (19.35%)** | **27 (87.1%)** | **3 (9.68%)** | **6 (19.35%)** | **25 (80.65%)** |
|  | **Age** | 35-45 | 5 | 4 (80%) | 1 (20%) | 5 (100%) | 0 | 2 (40%) | 3 (60%) |
|  |  | 45-55 | 14 | 10 (71.4%) | 2 (14.29%) | 8 (57.14%) | 1 (7.14%) | 2 (14.29%) | 12 (85.71%) |
|  |  | 55-65 | 9 | 5 (55.56%) | 3 (33.33%) | 7 (77.78%) | 2 (22.22%) | 1 (11.11%) | 8 (88.89%) |
|  |  | 65-75 | 3 | 1 (33.33%) | 0 | 2 (66.67%) | 0 | 1 (33.33%) | 2 (66.67%) |
| **Average** | | |  | **20 (64.52%)** | **6 (19.35%)** | **27 (87.1%)** | **3 (9.68%)** | **6 (19.35%)** | **25 (80.65%)** |
|  | **Tumor Grade** | Well differentiated | 9 | 6 (66.67%) | 2 (22.22%) | 8 (88.89%) | 1 (11.11%) | 2 (22.22%) | 7 (77.78%) |
|  |  | Moderately differentiated | 11 | 5 (45.45%) | 3 (27.27%) | 9 (81.82%) | 2 (18.18%) | 3 (27.27%) | 8 (72.73%) |
|  |  | Poorly differentiated | 11 | 9 (81.82%) | 1 (9.1%) | 10(90.90%) | 0 | 1 (9.1%) | 10 (90.90%) |
| **Average** | | |  | **20 (64.52%)** | **6 (19.35%)** | **27 (87.1%)** | **3 (9.68%)** | **6 (19.35%)** | **25 (80.65%)** |
|  | **Tumor Stage** | II | 2 | 1 (50%) | 1 (50%) | 2 (100%) | 0 | 1 (50%) | 1 (50%) |
|  |  | III | 3 | 2 (66.67%) | 1 (33.33%) | 2 (66.67%) | 1 (33.33%) | 1 (33.33%) | 2 (66.67%) |
|  |  | IV | 3 | 2 (66.67%) | 0 | 3(100%) | 0 | 1 (33.33%) | 2 (66.67%) |
|  |  | Unknown | 23 | 15 (65.22%) | 4 (17.39%) | 20 (86.96%) | 2 (8.7%) | 3 (13.04%) | 20 (86.96%) |
| **Average** | | |  | **20 (64.52%)** | **6 (19.35%)** | **27 (87.1%)** | **3 (9.68%)** | **6 (19.35%)** | **25 (80.65%)** |
|  | **Clinical staging** | Alveolus | 9 | 4 (44.44%) | 2 (22.22%) | 7 (77.78%) | 1 (11.11%) | 0 | 9 (100%) |
|  |  | Buccal | 8 | 7 (87.5%) | 1 (12.5%) | 7 (87.5%) | 1 (12.5%) | 0 | 8 (100%) |
|  |  | Gingiva | 7 | 4 (57.14%) | 2 (28.6%) | 6 (85.7%) | 1 (14.3%) | 3 (42.86%) | 4 (57.14%) |
|  |  | Alveolus & Buccal | 7 | 5 (71.4%) | 1 (14.3%) | 7 (100%) | 0 | 3 (42.86%) | 4 (57.14%) |
| **Average** | | |  | **20 (64.52%)** | **6 (19.35%)** | **27 (87.1%)** | **3 (9.68%)** | **6 (19.35%)** | **25 (80.65%)** |

| S.No. | Groups | | Total (*N*=31) | **STAT5B** | | **LRP1** | |
| --- | --- | --- | --- | --- | --- | --- | --- |
|  |  |  |  | Expression | Overexpression | Expression | Overexpression |
|  | **Sex** | M | 22 | 15(68.18%) | 6 (27.27%) | 13 | 2 |
|  |  | F | 9 | 8 (88.89%) | 1 (11.11%) | 3 (33.33%) | 1 (11.11%) |
| **Average** | | |  | **23(74.19%)** | **7 (22.58%)** | **16(51.61%)** | **3(9.68%)** |
|  | **Age** | 35-45 | 5 | 4 (80%) | 1 (20%) | 5 (100%) | 0 |
|  |  | 45-55 | 14 | 11 (78.57%) | 3 (21.4%) | 5 (35.71%) | 2 (14.29%) |
|  |  | 55-65 | 9 | 6 (66.67%) | 3 (33.33%) | 4 (44.44%) | 1 (11.11%) |
|  |  | 65-75 | 3 | 2 (66.67%) | 0 | 2 (66.67%) | 0 |
| **Average** | | |  | **23(74.19%)** | **7 (22.58%)** | **16(51.61%)** | **3(9.68%)** |
|  | **Tumor Grade** | Well differentiated | 9 | 4 (44.44%) | 4 (44.44%) | 7 (77.78%) | 0 |
|  |  | Moderately differentiated | 11 | 9 (81.82%) | 2 (18.18%) | 5 (45.45%) | 2 (18.18%) |
|  |  | Poorly differentiated | 11 | 10(90.90%) | 1 (9.1%) | 4 (36.36%) | 1 (9.1%) |
| **Average** | | |  | **23(74.19%)** | **7(22.58%)** | **16(51.61%)** | **3(9.68%)** |
|  | **Tumor Stage** | II | 2 | 2 (100%) | 0 | 2 (100%) | 0 |
|  |  | III | 3 | 2 (66.67%) | 1 (33.33%) | 2 (66.67%) | 1 (33.33%) |
|  |  | IV | 3 | 1 (33.33%) | 2 (66.67%) | 2 (66.67%) | 0 |
|  |  | Unknown | 23 | 18(78.26%) | 4 (17.39%) | 10(43.48%) | 2 (8.7%) |
| **Average** | | |  | **23(74.19%)** | **7(22.58%)** | **16(51.61%)** | **3(9.68%)** |
|  | **Clinical staging** | Alveolus | 9 | 7 (77.78%) | 1 (11.11%) | 3 (33.33%) | 1 (11.11%) |
|  |  | Buccal | 8 | 6 (75%) | 2 (25%) | 3 (37.5%) | 1 (12.5%) |
|  |  | Gingiva | 7 | 5 (71.4%) | 2 (28.6%) | 4 (57.14%) | 1 (14.3%) |
|  |  | Alveolus & Buccal | 7 | 5 (71.4%) | 2 (28.6%) | 6 (85.7%) | 0 |
| **Average** | | |  | **23(74.19%)** | **7(22.58%)** | **16(51.61%)** | **3(9.68%)** |

**Supplementary Table 2.** Gender based comparative analysis of % apoptotic priming and drug-response linked gene expression

| **Method** | **Male** | **Female** | **Interpretation** | **F-test (p-value)** |
| --- | --- | --- | --- | --- |
| Count | 22 | 9 | The tumor ratio of male and female was approximately 2:1 | NA |
| Average of 11 Gene Expression | 2.24 | 1.50 | Gene over expression was higher in male population than female population | Male Vs Female  0.07987 |
| Average of Δ Priming % | 52.22 | 57.15 | Average Priming of female was more than the Priming of male | Male Vs Female  0.7463 |
| Correlation: Gene expression Vs Δ Priming % | -0.7222 | -0.532 | The association Gene expression Vs Δ Priming % of female was higher compared to male in the difference of 19%. | NA |

**Supplementary Scheme 1**: Calculations

$\mathrm{BPD}={armax}_{i\epsilon\left\{ 1,2..31 \right\},j\epsilon\{1,2..5\}}\left( Drug\_Response[i,j] \right)$ (1)

${\mathrm{RD}_{\mathrm{ij}}}_{i\epsilon\left\{ 1,2..31 \right\},j\epsilon\{1,2..5\}}=Response({BPD}_{i})-Drug\_Response[i,j]$ (2)

where, *i* is the index of tumors and *j* is the index of drugs.

Example 1: For the first tumor, % apoptotic priming of paclitaxel was higher. So Best Priming Drug for the first tumor was paclitaxel.

We applied equation (2) to the first tumor sample by using the priming of all drugs to get RD values.

Paclitaxel- BPD: (41.237-41.237) = 0

Vinblastine – BPD: (41.237-37.957) =3.28

Doxorubicin – BPD: (41.237- 33.063) =8.174

Daunarubicin – BPD: (41.237-35.887) =5.35

Vincristine - BPD: (41.237-30.713) =10.524

The Relative Difference values were 0, 3.28, 8.174, 5.35 and 10.524

Example 2: For the fourth tumor, priming % of vinblastine was higher. So Best Priming Drug for fourth tumor is Vinblastine.

Apply Equation (2) to the fourth tumor using the priming of all drugs to get RD values.

Paclitaxel- BPD: (73.937-73.83) =0.107

Vinblastine – BPD (73.937-73.937) =0

Doxorubicin – BPD (73.937- 73.02) =0.917

Daunarubicin – BPD (73.937-71.64) =2.297

Vincristine -BPD (73.937-67.843) =6.094

The Relative Difference values were 0.107, 0, 0.917, 2.297 and 6.094

Among five drugs, paclitaxel (for 25 tumors) and vinblastine (for 6 tumors) gave higher priming; hence they were the best drugs. As in the example 2, the tumors 4, 6, 8, 19, 22 and 29 had higher priming by vinblastine and for each drug the priming difference with vinblastine was calculated. As in the example 1, the rest of the tumors had higher priming by paclitaxel for each drug the priming difference with paclitaxel was calculated. These differences in priming and the drug wise average of RD (last row) are given in the following table. The average response difference (RD) of the second ranking drug (vinblastine) with paclitaxel was 2.3% in these 25 tumor samples. The RD of paclitaxel with vinblastine in these 6 tumors was only 0.77%. Daunorubicin was ranked next in efficacy with a RD of 5.54% from paclitaxel. We observed that there was a larger RD between paclitaxel and doxorubicin (7.61%) and vincristine (10.9%) in the tumor samples (Fig. 4).

| Tumor | Paclitaxel | Vinblastin | Doxorubicin | Daunarubicin | Vincristin |
| --- | --- | --- | --- | --- | --- |
| 1 | 0 | 3.28 | 8.173333 | 5.35 | 10.52333 |
| 2 | 0 | 4.996667 | 7.526667 | 6.366667 | 10.71333 |
| 3 | 0 | 2.766667 | 7.676667 | 6.213333 | 12.53 |
| 4 | 0.106667 | 0 | 0.916667 | 2.296667 | 6.093333 |
| 5 | 0 | 5.6 | 8.136667 | 4.54 | 9.903333 |
| 6 | 1.366667 | 0 | 5.77 | 5.93 | 10.89 |
| 7 | 0 | 2.813333 | 8.386667 | 5.33 | 10.01 |
| 8 | 2.486667 | 0 | 7.923333 | 8.623333 | 16.91667 |
| 9 | 0 | 1.07 | 7.136667 | 5.206667 | 9.5 |
| 10 | 0 | 3.116667 | 5.69 | 6.023333 | 8.29 |
| 11 | 0 | 4.183333 | 8.403333 | 6.74 | 11.84667 |
| 12 | 0 | 0.363333 | 6.78 | 4.106667 | 13.18 |
| 13 | 0 | 1.333333 | 7.526667 | 5.03 | 12.20667 |
| 14 | 0 | 2.413333 | 8.476667 | 5.486667 | 10.92667 |
| 15 | 0 | 1.253333 | 8.34 | 5.103333 | 13.62 |
| 16 | 0 | 2.246667 | 5.87 | 5.016667 | 10.43667 |
| 17 | 0 | 3.02 | 8.096667 | 5.7 | 10.41333 |
| 18 | 0 | 4.016667 | 8.006667 | 5.783333 | 10.93333 |
| 19 | 0.293333 | 0 | 5.333333 | 2.676667 | 7.233333 |
| 20 | 0 | 1.766667 | 7.586667 | 4.56 | 9.06 |
| 21 | 0 | 0.866667 | 9.896667 | 5.74 | 14.67 |
| 22 | 0.04 | 0 | 3.09 | 2.553333 | 9.233333 |
| 23 | 0 | 3.25 | 8.596667 | 7.84 | 10.11333 |
| 24 | 0 | 2.306667 | 7.59 | 4.326667 | 9.31 |
| 25 | 0 | 3.523333 | 11.30667 | 8.006667 | 13.21667 |
| 26 | 0 | 8.37 | 11.88667 | 8.723333 | 12.15333 |
| 27 | 0 | 2.556667 | 7.933333 | 6.81 | 9.686667 |
| 28 | 0 | 1.64 | 7.466667 | 4.266667 | 8.493333 |
| 29 | 1.2 | 0 | 10.3 | 6.486667 | 15.11667 |
| 30 | 0 | 1.626667 | 7.273333 | 4.763333 | 10.70667 |
| 31 | 0 | 3.103333 | 8.823333 | 6.163333 | 10.22 |
| **Average** | **0.177204** | **2.305914** | **7.610323** | **5.540753** | **10.90796** |

**Supplementary Figure Legends**

**Supplementary Figure 1:** Schematic representation for predicting drug response in oral tumor samples and experimental cell lines. (**A**)The tissue biopsies obtained were divided into two parts, one part for gene expression analysis and the other part for BH3 profiling. Gene expression analysis was performed to determine the relative expression pattern of genes linked to drug resistance. The BH3 profiling was determined based on mitochondrial apoptotic priming. The gene expression analysis and percentage of apoptotic priming analysis were further correlated then the drug response could be predicted. (**B**) The cells were treated with different chemotherapeutic drugs (1 µM). Further cell-based analyses were performed to finally predict drug response.

**Supplementary Figure 2:** Mass spectrometry analysis (MALDI-TOF MS) of the BIM used for BH3 profiling.

**Supplementary Figure 3:** High Performance Liquid Chromatography (HPLC) analysis of the synthesized BIM peptide employed in this study for the analysis of BH3 profiling.

**Supplementary Figure 4:** Hematoxylin and Eosin staining of tumor samples. Based on the cell architecture, the tissues were classified into different grades. Arbitrarily collected normal sample shows intact architectures. Tumor sample 9 shows clear differentiation, tumor sample 5 shows moderate differentiation and tumor sample 21 shows poor differentiation.

**Supplementary Figure 5:** Range of gene expression pattern of 11 genes based on their Ct values.

**Supplementary Figure 6:** Cluster plot of Ct-values of drug response linked genes and the % apoptotic priming of the chemotherapeutic drugs.

**Supplementary Figure 7:** Apoptotic priming potential of different anticancer drugs in OSCC primary cells. About 40-45% of apoptotic priming was observed in primary cells isolated from tumor biopsies by paclitaxel. Vinblastine and daunorubicin show 40% apoptotic priming, whereas doxorubicin and vincristine showed only 30-35% apoptotic priming.

**Supplementary Figure 8:** Linear and polynomial regression analysis of MDR-linked gene expression with paclitaxel-induced apoptotic priming. The uncertainty in this linear relationship suggests that the correlation between MDR gene expression and paclitaxel-induced apoptotic priming might also depend on other factors such as age, sex, tumor grade and tumor stage.

**Supplementary Table 1:** Gene expression pattern of 11 genes (ABCB1, ABCG2, ABCC1, ABCC2, ABCC3, ABCC5, CDKN1A, CDK2, STAT5B, LRP1 and TP53) in OSCC tissue samples with their clinical and pathological criteria. The gene expression levels were analyzed in three independent experiments.

**Supplementary Table 2:** Gender based comparative analysis of apoptotic priming and gene expression. The table provides the evidence for the existence of association between sex and gene expression with sex and priming % were calculated by taking average and correlation.

**Supplementary Scheme 1:** The elaborate explanation for the equation 1 and 2 was given along with the examples to show the way in which the BPD and RD of drugs were arrived.
